# CD81 is dispensable for hepatitis C virus cell-to-cell transmission in hepatoma cells

**DOI:** 10.1099/vir.0.006700-0

**Published:** 2009-01

**Authors:** Jeroen Witteveldt, Matthew J. Evans, Julia Bitzegeio, George Koutsoudakis, Ania M. Owsianka, Allan G. N. Angus, Zhen-Yong Keck, Steven K. H. Foung, Thomas Pietschmann, Charles M. Rice, Arvind H. Patel

**Affiliations:** 1 MRC Virology Unit, Institute of Virology, University of Glasgow, Church Street, Glasgow G11 5JR, UK; 2 Center for the Study of Hepatitis C, The Rockefeller University, New York, NY, USA; 3 Twincore Center for Experimental and Clinical Infection Research, Department of Experimental Virology, Hannover, Germany; 4 Helmholtz Center for Infection Research, Braunschweig, Germany; 5 Medizinische Hochschule Hannover, Germany; 6 Okairòs-Ceinge, Naples, Italy; 7 Department of Pathology, Stanford University School of Medicine, Stanford, CA 94305, USA

## Abstract

Hepatitis C virus (HCV) infects cells by the direct uptake of cell-free virus following virus engagement with specific cell receptors such as CD81. Recent data have shown that HCV is also capable of direct cell-to-cell transmission, although the role of CD81 in this process is disputed. Here, we generated cell culture infectious strain JFH1 HCV (HCVcc) genomes carrying an alanine substitution of E2 residues W529 or D535 that are critical for binding to CD81 and infectivity. Co-cultivation of these cells with naïve cells expressing enhanced green fluorescent protein (EGFP) resulted in a small number of cells co-expressing both EGFP and HCV NS5A, showing that the HCVcc mutants are capable of cell-to-cell spread. In contrast, no cell-to-cell transmission from JFH1_ΔE1E2_-transfected cells occurred, indicating that the HCV glycoproteins are essential for this process. The frequency of cell-to-cell transmission of JFH1_W529A_ was unaffected by the presence of neutralizing antibodies that inhibit E2–CD81 interactions. By using cell lines that expressed little or no CD81 and that were refractive to infection with cell-free virus, we showed that the occurrence of viral cell-to-cell transmission is not influenced by the levels of CD81 on either donor or recipient cells. Thus, our results show that CD81 plays no role in the cell-to-cell spread of HCVcc and that this mode of transmission is shielded from neutralizing antibodies. These data suggest that therapeutic interventions targeting the entry of cell-free HCV may not be sufficient in controlling an ongoing chronic infection, but need to be complemented by additional strategies aimed at disrupting direct cell-to-cell viral transmission.

## INTRODUCTION

Hepatitis C virus (HCV) is a positive-strand RNA virus, whose entry into host cells depends on the interaction of its envelope proteins E1 and E2 with several cellular receptors. In recent years, the mechanisms of HCV entry have been largely investigated using retrovirus-based pseudoparticles bearing HCV envelope proteins (HCVpp), and latterly with the newly developed authentic HCV cell culture infection (HCVcc) system (Helle & Dubuisson, 2008; von Hahn & Rice, 2008). Both the HCVpp and HCVcc systems support a crucial role for CD81 in mediating virus infection. CD81 is a plasma membrane protein containing a small and a large extracellular loop (LEL), the latter of which mediates binding to E2. Infectivity assays have shown that it is necessary but not sufficient for virus entry into hepatocytes (Helle & Dubuisson, 2008; von Hahn & Rice, 2008). Recent studies have identified several conserved E2 residues located within discontinuous regions as essential for E2–CD81 interaction and virus entry (Drummer *et al.*, 2006; Owsianka *et al.*, 2006; Rothwangl *et al.*, 2008). Interestingly, some of these residues (W420, Y527, W529, G530 and D535) also contribute to the epitopes of various broadly neutralizing antibodies (nAbs) (Keck *et al.*, 2008; Law *et al.*, 2008; Owsianka *et al.*, 2008; Perotti *et al.*, 2008; Tarr *et al.*, 2006).

In addition to entry via receptor-mediated endocytosis, HCV has recently been shown to spread by direct cell-to-cell transfer. Valli *et al.* (2006, 2007) provided evidence of direct virus transfer into HepG2 cells upon co-cultivation with persistently infected lymphoblastoid B-cells. Similarly, cell-to-cell transmission of wild-type (WT) HCVcc was shown to occur upon co-cultivation of infected hepatoma cells with their naïve counterparts under subneutralizing conditions (Timpe *et al.*, 2008). The latter study suggested that this route of virus transmission may be independent of CD81. Conversely, a more recent report described a CD81-deficient cell line that was resistant to infection with exogenously added HCVcc, and to cell-to-cell transmission of virus, concluding that CD81 is absolutely required for this process (Russell *et al.*, 2008).

We aimed to resolve this issue by using HCVcc mutants defective in CD81 binding and cell entry, and hepatoma-derived cell lines deficient in CD81 expression. Our results show that the mutant HCVcc are capable of cell-to-cell transmission in cultured cells and provide strong evidence that CD81 is not required for this process.

## METHODS

### Antibodies.

Mouse monoclonal antibody (mAb) AP33, and the human mAbs CBH-4B and -7, all recognizing HCV E2, and the anti-NS5A mouse mAb 9E10 have been described previously (Clayton *et al.*, 2002; Hadlock *et al.*, 2000; Lindenbach *et al.*, 2005). The sheep antiserum to NS5A was a kind gift from M. Harris (Institute of Molecular and Cellular Biology, University of Leeds, Leeds, UK). The production of the rabbit polyclonal anti-E1 antiserum R1233 will be described elsewhere. The murine leukemia virus (MLV) gag-specific mAb was obtained from rat hybridoma cells (CRL-1912; ATCC). The anti-CD81 mAb (clone JS-81) was purchased from BD Pharmingen.

### Cell lines.

Human hepatoma Huh7 and related cells, and human epithelial kidney (HEK)-293T cells were grown in Dulbecco's modified Eagle's medium supplemented with 10 % fetal calf serum. Huh7-Lunet (Huh7L) cells represent a subclone of Huh7 cells highly permissive for HCV RNA replication and display a bimodal expression level of CD81 (Friebe *et al.*, 2005; Koutsoudakis *et al.*, 2007). Huh7L cells expressing high levels of CD81 (Huh7L-H/EF) were obtained by stable transfection with pEF1/V5-HisA/CD81 (Koutsoudakis *et al.*, 2007), whereas cells with low endogenous CD81 expression were derived by sorting the Huh7L cell population for cells expressing little or no CD81 (Huh7L-N). The Huh7L-N cells were subsequently subjected to limiting dilution cloning. Individual subclones, Lunet/CD81N#4 (Huh7L-#4) and Lunet/CD81N#7 (Huh7L-#7), expressing very low or barely detectable levels of CD81, were identified by flow cytometry.

To generate stable short hairpin RNA (shRNA)-expressing cells, Huh7.5 cells (Blight *et al.*, 2002) were transduced with VSV-Gpp packaged shRNA expression vector pLenti-3′-U6-EC-EP7, a human immunodeficiency virus type 1 provirus expressing the shRNA of interest from an internal U6 promoter and the blasticidin selectable marker from a second promoter (kind gift from Daniel Boden, The Aaron Diamond AIDS Research Center, New York, USA) (MacDonald *et al.*, 2007). Nucleotide sequences expressing shRNA targeting the CD81 sequence 5′-ACCTGCTCTTCGTCTTCAATT-3′ or 5′-ACCTCAGTGCTCAAGAACA-3′ (nt 268–288 or 732–750 relative to the CD81 cDNA sequence, respectively) were cloned into pLenti-3′-U6-EC-EP7 to generate pLenti-3′-CD81sh268 or pLenti-3′-CD81sh732. Huh7.5 cells stably expressing these shRNAs were designated Huh7.5-268 or Huh7.5-732, respectively. The pLenti-3′-irrelevant-shRNA, which is not predicted to target any human or murine sequences, was used to generate negative-control shRNA cells (Huh7.5-irrshRNA) (MacDonald *et al.*, 2007). Transduced Huh7.5 cells were selected by using 6 μg blasticidin ml^−1^ and the surviving cells were pooled and used in experiments.

Huh7.5-268 cells were complemented for CD81 expression by transduction with VSV-Gpp packaged lenti-provirus expressing a green fluorescent protein (GFP)–CD81 fusion in which the sh268 targeted sequence was disrupted by incorporating silent mutations (shown in lower case, TAttTatTaTTtGTgTTtAAc) by using overlapping PCR. This mutant CD81 open reading frame was cloned in-frame downstream of the GFP sequence in TRIP–GFP (Zennou *et al.*, 2000) creating a fusion protein with CD81 (Huh7.5-268–hCD81). The same was done with the cell line transduced with pLenti-3′-irrelevant-shRNA (Huh7.5-irrshRNA–hCD81). TRIP–GFP-expressing cells were used as negative control. Huh7.5 cells were transduced at an m.o.i. greater than 10, such that essentially all cells in the population expressed the transgene (confirmed by FACS analysis, data not shown).

To use as GFP-expressing recipient cells in cell-to-cell transfer assays, all Huh7-, Huh7L- and Huh7.5-derived cell lines were transduced with a retrovirus vector carrying enhanced green fluorescent protein (EGFP), followed by selection in medium supplemented with 300 μg G418 ml^−1^.

### HCVpp infection assay.

The cDNA sequence encoding the HCV genotype 2a strain JFH1 (Wakita *et al.*, 2005) aa 132–746 representing the C terminus of core, E1 and E2 were cloned into the mammalian expression vector phCMV. Alanine substitution of W529 and D535 was performed by fusion PCR using appropriate primers and the mutated sequences verified by nucleotide sequencing. The plasmids expressing the MLV Gag–Pol, and the MLV transfer vector expressing GFP were gifts from Francois-Loic Cosset (Inserm U758 – Ecole Normale Supérieure de Lyon, Lyon Cedex 07, France). HCVpp were produced as described previously (Bartosch *et al.*, 2003; Owsianka *et al.*, 2005). The medium containing HCVpp was collected, clarified, filtered through a 0.45 μm pore membrane, and subjected to ultracentrifugation through a 20 % sucrose cushion at 116 000 ***g*** for 2 h at 4 °C.

### Generation of HCVcc virus.

The plasmids pJFH1 containing the full-length cDNA and one lacking E1E2 sequences (pJFH1/ΔE1E2) of the HCV genotype 2a strain were a kind gift from Takaji Wakita (Wakita *et al.*, 2005). The mutant E2 sequences were subcloned from the phCMV plasmid described above to pJFH1. The *Renilla* luciferase-expressing HCVcc, FL-J6/JFH-5′C19Rluc2AuUbi, has been described previously (Tscherne *et al.*, 2006). HCVcc were generated essentially as described previously (Wakita *et al.*, 2005). Briefly, linearized plasmids carrying HCVcc genomic cDNA were used as a template to generate viral genomic RNA by *in vitro* transcription. Ten micrograms of this RNA was electroporated into Huh7 cells. At 4 days post-transfection, the culture medium was harvested, filtered through 0.45 μm pore membrane and used as virus stock in infection assays. Naïve Huh7 cells were infected with virus stocks for 3 h and fixed with methanol for immunofluorescence (IF) assays as described below or lysed in Trizol LS (Invitrogen) for total RNA extraction.

### Sucrose density-gradient ultracentrifugation analysis of HCVcc and viral RNA quantification.

Virus particles released from transfected cells were concentrated by centrifugation through a sucrose cushion as described above, and then subjected to a 20–50 % (w/w) linear sucrose gradient centrifugation at 116 000 ***g*** for 16 h at 4 °C. One millilitre fractions were collected and the viral RNA concentration was determined by absolute quantification by using a real-time RT-PCR (RT-qPCR) assay. *In vitro*-transcribed HCV strain JFH1 genomic RNA of known concentration was used as a standard. The probe sequence used was 6-FAM-AAAGGCCTTGTGGTACTG-MGB (Applied Biosystems), and primer sequences were: forward, 5′-TCTGCGGAACCGGTGAGTAC-3′ and reverse, 5′-GCACTCGCAAGCGCCCTATC-3′ (Sigma).

Viral RNA levels in infected cells were quantified in a relative RT-qPCR. Samples were run as a multiplex reaction containing the primers and FAM probe described above, and a pre-validated human glyceraldehyde-3-phosphate dehydrogenase endogenous control primer/probe mix. The values obtained were normalized to WT.

### Detection of cell-to-cell transport.

To detect cell-to-cell transfer of HCV, freshly electroporated Huh7 cells were seeded and incubated for a 24 h period, and then washed with medium to remove residual RNA before adding the EGFP-expressing recipient cells. The co-cultured cells were grown to confluency, trypsinized and reseeded on coverslips for IF analysis or fixed with 1 % paraformaldehyde and permeabilized with 0.1 % saponin for FACS analysis. The cells were stained using sheep anti-NS5A followed by a tetramethyl rhodamine isothiocyanate (TRITC)-conjugated secondary antibody for IF or the anti-NS5A mAb 9E10 followed by a phycoerythrin (PE)-conjugated secondary antibody for FACS analysis.

### GNA (*Galanthus nivalis* antigen) capture assay for E2 analysis.

ELISAs to detect mAb binding to E2 glycoprotein in transfected HEK-293T and Huh7 cells were performed essentially as described previously (Patel *et al.*, 2000). Bound glycoproteins were detected using the anti-E2 mAbs AP33, CBH-4B or -7, followed by an anti-species IgG–horseradish peroxidase (HRP) and 3,3′,5,5′-tetramethylbenzidine (TMB) substrate. Absorbance values were determined at 450 nm.

### Indirect IF.

To examine intracellular expression of HCV proteins, cells on coverslips were fixed in methanol, washed with PBS containing 0.05 % Tween-20 (PBS-T) and incubated at room temperature for 1 h with specific antibodies to NS5A. Cells were washed with PBS-T, stained with anti-species IgG conjugated with either fluorescein isothiocyanate or TRITC (Sigma) for 1 h, washed with PBS-T, and mounted on a glass slide and examined with a Zeiss Laser Scanning Microscope.

### E2–CD81-binding assay.

Human CD81-LEL fused to glutathione *S*-transferase (GST–CD81) was expressed in *Escherichia coli* and bound to glutathione–agarose beads (Sigma). After equilibration with cell lysis buffer (20 mM Tris pH 7.5, 1 mM EDTA, 150 mM NaCl, 20 mM iodoacetamide, 1 % NP-40), lysates of Huh7 cells electroporated with viral RNA were added and incubated for 30 min. As a control, an unrelated GST fusion protein was used as bait. The beads were washed three times with lysis buffer and the pull-down products analysed by Western immunoblots as described previously (Owsianka *et al.*, 2005).

## RESULTS

### Expression and analysis of mutated HCV strain JFH1 E2

**Fig. 1. f1:**
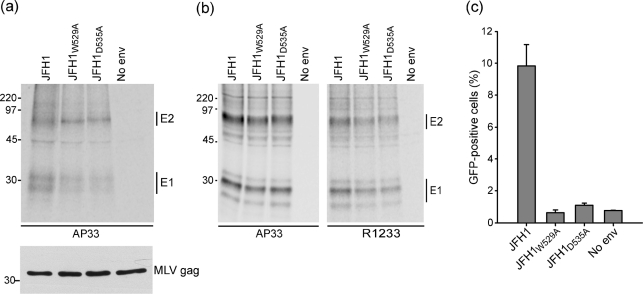
Heterodimerization and functional analysis of mutated JFH1 E2. HEK-293T cells co-transfected with MLV packaging and transfer vectors together with a construct expressing HCV JFH1 WT or mutant E2 were radiolabelled with [^35^S]methionine as described previously (Owsianka *et al.*, 2005). Viral glycoproteins present in (a) the secreted HCVpp and (b) the cell lysates were immunoprecipitated using anti-E1 antiserum R1233 or the anti-E2 mAb AP33 as shown and the immune complexes analysed by non-reducing SDS-PAGE. The radiolabelled proteins were visualized using a Bio-Rad Personal FX phosphorimager. The HCVpp were also subjected to Western immunoblotting using a MLV gag-specific mAb to detect gag proteins (bottom of panel a). The positions of molecular mass markers in kDa are shown. (c) HCVpp incorporating the WT or mutated JFH1 E2 were used to infect Huh7 cells. The infectivity, expressed as GFP-positive cells (%), was measured as described in the Methods.

We investigated the functional importance of the conserved E2 residues W529 and D535 (previously shown to be critical for HCV genotype 1a E2–CD81 interaction and HCVpp entry; Owsianka *et al.*, 2006) in the genotype 2a strain JFH1. The corresponding residues in JFH1 are W531 and D537 (Kato *et al.*, 2001), but we designated them as W529 and D535 in keeping with the recommendation to assign absolute amino acid numbering, based on the reference genotype 1a strain H77 polyprotein (Yanagi *et al.*, 1997), to the HCV proteins of different genotypes (Kuiken *et al.*, 2006). The consequence of substituting each of these residues with alanine was studied initially using the HCVpp system. Immunoprecipitation using anti-E1 and anti-E2 antibodies showed that the mutants formed non-covalent E1E2 heterodimers as efficiently as the WT proteins in both HCVpp and cell lysates (Fig. 1a and b[Fig f1]), but HCVpp infectivity was completely abrogated (Fig. 1c[Fig f1]).

**Fig. 2. f2:**
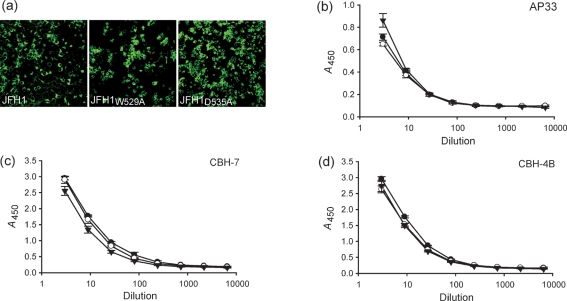
Characterization of mutant HCVcc. (a) Huh7 cells electroporated with WT or mutant HCVcc RNA were incubated for 4 days after which they were fixed, stained for NS5A and analysed by confocal microscopy. (b–d) Serial dilutions of Huh7 cell lysates from (a) were analysed for E2 levels by GNA capture ELISA using mouse mAb AP33 that recognizes a linear epitope (Clayton *et al.*, 2002), or the conformation-dependent human mAbs CBH-7 and -4B (Hadlock *et al.*, 2000). The mean absorption values with sd bars derived from experiments performed in triplicate are shown. •, WT JFH1; ○, JFH1_W529A_; ▾, JFH1_D535A_.

We next introduced the W529A or D535A substitution into the JFH1 genomic cDNA for analysis in the HCVcc system. The viral NS5A protein was expressed at comparable levels in cells electroporated with WT and mutant RNA transcripts, indicating that the mutations in E2 had no apparent effect on viral RNA replication (Fig. 2a[Fig f2]). Furthermore, the levels of mutant E2 in these cells were similar to that of the WT glycoprotein, as detected using anti-E2 mAbs recognizing linear (Clayton *et al.*, 2002) or conformation-dependent epitopes (Hadlock *et al.*, 2000) (Fig. 2b–d[Fig f2]). Thus, the mutations had no discernible effect on the expression or overall conformation of E2 in the HCVcc system.

### Strain JFH1 E2 residues W529 and D535 are critical for CD81 binding and virus infectivity but not for virus particle assembly

**Fig. 3. f3:**
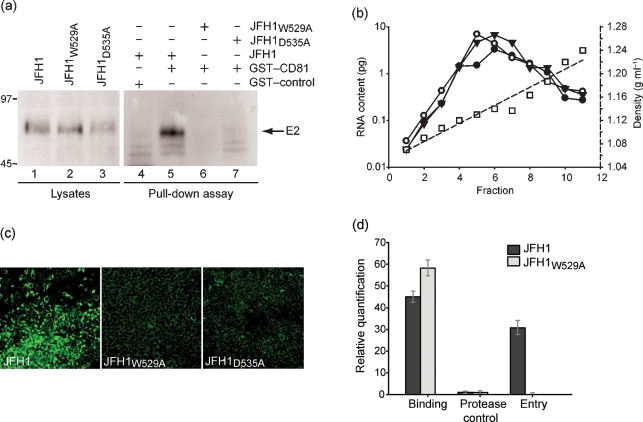
Alanine substitution of E2 residues W529 or D535 abrogate E2–CD81 interaction and HCVcc infectivity, but not virus particle assembly and secretion. (a) Lysates of Huh7 cells electroporated with WT or mutant HCVcc RNA were incubated with GST–CD81 or an irrelevant GST-fusion protein bound to glutathione–agarose beads in a pull-down assay. The resulting protein complexes (lanes 4–7) as well as the original lysates (lanes 1–3) were subjected to immunoblot using the anti-E2 mAb AP33. The positions of molecular mass markers in kDa are shown. (b) Sucrose cushion-pelleted HCVcc particles from culture medium of Huh7 cells electroporated with WT JFH1 (•), JFH1_W529A_ (▾) or JFH1_D535A_ (○) were subjected to a 20–50 % sucrose gradient centrifugation, then fractions were collected from the top of the gradient and the viral RNA content was determined. The sucrose density (□) in each fraction was also measured. (c) Culture medium of cells electroporated with WT or mutant HCVcc RNA was used to infect naïve Huh7 cells. Following incubation for 4 days, the cells were fixed and analysed for NS5A by confocal microscopy. (d) Mutant virus particles bind, but do not enter cells. Huh7 cells were incubated at 4 °C with sucrose cushion-concentrated virus particles for 2 h. The pre-incubated cells were then split three ways. The first batch of cells was washed with PBS and lysed for total RNA extraction. The second batch was washed three times, followed by incubation with 50 μg proteinase K ml^−1^ at 4 °C for 1 h with gentle agitation before lysing for RNA extraction. The third batch of cells was placed in fresh medium after removing the virus inoculum, shifted to 37 °C for 6 h and washed to remove any remaining cell surface-bound HCV particles before RNA extraction. The relative quantity of viral RNA in each of the total RNA samples was determined by RT-qPCR.

To test whether the E2 mutants were interacting with CD81, we performed a pull-down assay using a GST–CD81 fusion protein and lysates of Huh7 cells electroporated with viral RNA. Both mutant and WT E2 were expressed in cells in comparable amounts (Fig. 3a[Fig f3], lanes 1–3). No WT E2 band was detected when an unrelated GST-fusion protein was used as bait in the pull-down assay (lane 4). With the GST–CD81 beads, a clear signal for WT but not mutant E2 was observed, indicating that both the W529A and D535A substitutions in E2 indeed abrogated the interaction between the JFH1 glycoprotein and CD81 (lanes 5–7).

As the mutations are in a structural protein, we assessed whether HCVcc particles were assembled and secreted, and whether they differed from WT in their biophysical properties. Fractionation of HCVcc over a sucrose gradient showed that both the mutant and WT viral RNA sedimented at a density of 1.13–1.14 g ml^−1^ (Fig. 3b[Fig f3]), which is consistent with that reported previously for WT HCVcc (Lindenbach *et al.*, 2005; Wakita *et al.*, 2005). We conclude that alanine substitution of W529 or D535 in the genotype 2a JFH1 HCVcc has no effect on viral RNA replication nor on the production of virus particles and their biophysical properties. We then tested the ability of these particles to infect naïve cells. In contrast to the WT virus-infected cells, no NS5A expression was observed in cells infected with the mutant virus particles (Fig. 3c[Fig f3]). RT-qPCR analysis confirmed the absence of viral RNA in mutant virus-infected cells (data not shown). Thus, in keeping with our HCVpp results (Fig. 1c[Fig f1]), the substitution W529A or D535A in E2 completely abrogated HCVcc infectivity.

### Mutant virus particles do not enter cells

To find out whether the non-infectivity of the mutants was due to a block at cell binding or at a post-binding stage, Huh7 cells were incubated at 4 °C with virus particles for 2 h. Quantification of viral RNA on these cells showed that both mutant and WT viruses were able to efficiently bind cells (Fig. 3d[Fig f3]). To test for virus entry, cells incubated with virus particles at 4 °C for 2 h were shifted to 37 °C for 6 h and then treated with proteinase K to remove any remnant particles from the cell surface. After this treatment, viral RNA could be detected in cells incubated with the WT but not with the mutant virus, which indicates that the failure of the mutants to infect naïve cells is due to a defect at a post-binding step. As a control, cells pre-incubated with virus particles at 4 °C for 2 h were immediately treated with proteinase K and then tested for viral RNA. A very low signal was obtained with all three viruses, demonstrating that the proteinase K treatment efficiently removed cell-bound HCVcc particles (Fig. 3d[Fig f3]).

### Mutant viruses are competent for direct cell-to-cell spread

**Fig. 4. f4:**
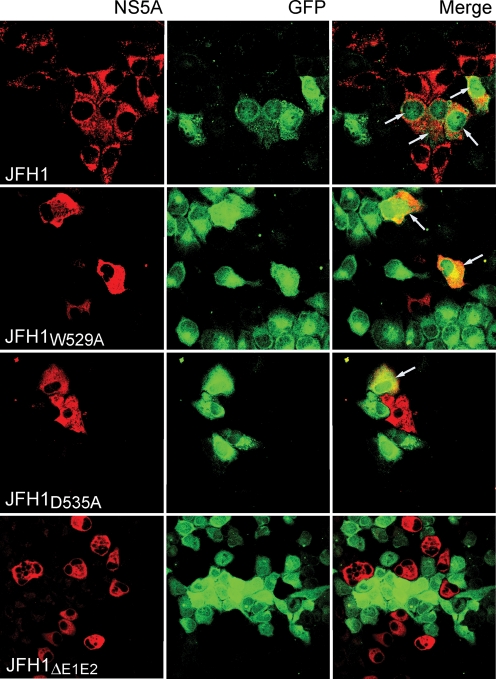
Cell-to-cell spread of HCVcc E2 mutants. Huh7 cells electroporated with WT or mutant JFH1 RNA were co-cultured with naïve Huh7–GFP. The cells were fixed in methanol, probed for NS5A using the anti-NS5A antiserum and anti-sheep IgG-TRITC conjugate, and examined with a Zeiss Laser Scanning Microscope. Arrows mark GFP-positive cells expressing NS5A as a result of virus cell-to-cell transmission.

There have been conflicting reports on the role of CD81 in direct cell-to-cell transmission of HCV. Using the HCVcc mutants described here, we investigated whether CD81 is necessary for cell-to-cell transmission of HCV in cultured cells. Huh7 cells electroporated with JFH1_W529A_ or JFH1_D535A_ RNA were grown for 24 h, then washed and co-cultured with naïve Huh7 cells constitutively expressing EGFP (Huh7–GFP). To maximize the opportunity for cell-to-cell interactions and virus transfer, the cells were grown to confluency, trypsinized, reseeded on coverslips and analysed the next day by IF for NS5A expression. Cells expressing both EGFP and HCV NS5A would be indicative of cell-to-cell transmission of virus from the electroporated Huh7 donor cells to the recipient Huh7–GFP cells. We observed a small number of cells expressing both EGFP and NS5A, indicating that both mutants were capable of cell-to-cell spread in tissue culture (Fig. 4). In contrast, co-cultivation of Huh7–GFP cells with Huh7 cells electroporated with JFH1_ΔE1E2_ RNA (lacking the viral E1E2 glycoprotein-encoding sequences) yielded no EGFP/NS5A-positive cells, suggesting that this mode of virus spread is E1E2-dependent (Fig. 4).

The ability of the CD81 binding defective viruses to spread via direct cell-to-cell transmission indicates that CD81 may not be required in this process. However, a possibility remains that the interaction between the mutant E2 and CD81, even though undetectable in our GST–CD81 pull-down assays, is not completely abrogated. Also, a low affinity of mutant E2 for CD81 may in part be compensated by cell-to-cell contact facilitating HCV–CD81 interactions, thus allowing a low level of entry to occur. Furthermore, although the CD81-binding mutants were shown to be non-infectious and no infectious particles were observed over multiple passages, the presence of infectious particles arising through complementary/revertant mutations cannot be excluded. However, co-cultivation experiments in the presence of a well-characterized neutralizing anti-CD81 mAb did not alter the levels of cell-to-cell transmission (Supplementary Fig. S1a and b available in JGV Online). These findings were further substantiated by co-cultivation experiments in the presence of neutralizing anti-E2 antibodies, known to block E2–CD81 interactions, which also did not affect levels of cell-to-cell transmission (Supplementary Fig. S1c).

**Fig. 5. f5:**
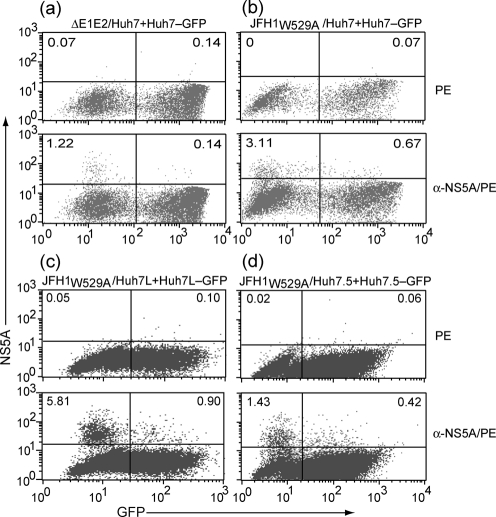
Quantitations of cell-to-cell spread of mutant HCVcc using flow cytometry. Huh7 cells electroporated with (a) JFH1_ΔE1E2_ or (b) JFH1_W529A_ RNA were co-cultured with Huh7–GFP cells. Following incubation, cells were fixed and probed with the anti-NS5A mAb 9E10 and analysed by FACS. Similar analysis was performed using (c) Huh7L or (d) Huh7.5 cells electroporated with JFH1_W529A_ RNA. The upper left quadrant of each dot plot shows the percentage of NS5A-expressing donor cells, and the upper right quadrant shows the percentage of recipient cells co-expressing EGFP and NS5A. The populations of untransfected and uninfected cells are shown in the lower left and lower right quadrants, respectively. The upper dot plot in each panel represents cells that have been probed with only the PE-conjugated secondary antibody to serve as negative control.

To quantify the levels of cell-to-cell viral transmission, the co-cultured cells were analysed by flow cytometry. The level of dually stained cells, and thus cell-to-cell transmission of JFH1_W529A_ was approximately 0.6 % of the total cell population (i.e. 16 % of all NS5A-positive cells) (Fig. 5b[Fig f5], lower panel). No cell-to-cell transmission was observed when Huh7 cells electroporated with a viral RNA lacking E1E2-encoding sequences were co-cultured with Huh7–GFP cells (Fig. 5a[Fig f5], lower panel), confirming that the viral glycoproteins are essential for this process. We next tested the ability of JFH1_W529A_ to spread by cell-to-cell transfer in two related cell lines, Huh7L and Huh7.5. In keeping with our data on Huh7 cells, the frequency of cell-to-cell transmission from Huh7L to Huh7L–GFP or from Huh7.5 to Huh7.5–GFP as the percentage of double-positive cells of all NS5A-positive cells was 12 and 20 %, respectively (Fig. 5c and d[Fig f5], lower panels).

### HCVcc cell-to-cell transmission is not dependent on CD81 expression

**Fig. 6. f6:**
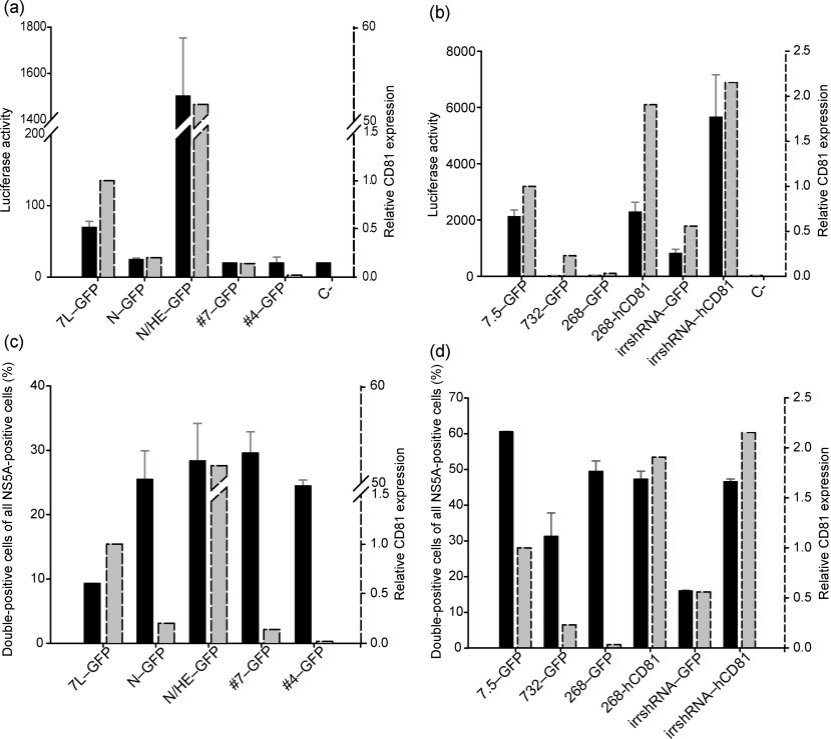
Cell-free virus infection, but not cell-to-cell transmission, correlates with CD81 expression. (a) Huh7L- or (b) Huh7.5-derived cell lines expressing varying levels of CD81 were infected with the luciferase-expressing virus, FL-J6/JFH-5′C19Rluc2AuUbi. At 3 days p.i., infectivity was measured by determining intracellular luciferase activity, which is presented as relative light units (black bars). In parallel, the relative levels of CD81 surface expression, presented as mean fluorescence intensity (grey bars), were determined by FACS using an anti-CD81 mAb and PE-conjugated secondary antibody. (c) Huh7L or (d) Huh7.5 cells electroporated with JFH1_W529A_ RNA were co-cultured with their corresponding Huh7L- or Huh7.5-derived CD81 variant cell lines. Following incubation for 4 days, cells were fixed and analysed by FACS. The frequency of cell-to-cell spread is presented as double-positive (NS5A/GFP) of all NS5A-positive cells (%) (black bars). The relative levels of CD81 expression (as derived in a and b above) in each cell line are plotted side by side (grey bars).

To further confirm the dispensability of CD81 in HCVcc cell-to-cell transmission, we generated stable Huh7L- and Huh7.5-derived cell lines that express little or no CD81 (N–GFP, #4–GFP, #7–GFP, 268–GFP and 732–GFP), or elevated levels of CD81 (N/HE–GFP, 268hCD81–GFP, irrshRNA–hCD81). We also engineered them to stably express EGFP to use them as recipients in our cell-to-cell transfer assay. The EGFP-expressing cell lines were first tested for susceptibility to HCVcc infection using the FL-J6/JFH-5′C19Rluc2AuUbi virus that expresses luciferase (Tscherne *et al.*, 2006). As expected, there was a clear correlation between CD81 expression and virus infectivity (Fig. 6a and b[Fig f6]). Lines expressing low levels of CD81 were refractive to infection and showed only background levels of luciferase activity, in keeping with previous studies which show that productive HCVcc infection requires a minimal level of CD81 expression (Koutsoudakis *et al.*, 2007).

We next investigated the cell-to-cell transmission of JFH1_W529A_ HCVcc in these cell lines. Parent Huh7L and Huh7.5 cell lines electroporated with JFH1_W529A_ RNA were co-cultured with their corresponding (EGFP-expressing) CD81-deficient or CD81-overexpressing counterparts and analysed by flow cytometry. We found that the CD81 status of the recipient cells had no influence on the frequency of cell-to-cell virus transmission (Fig. 6c and d[Fig f6]).

**Fig. 7. f7:**
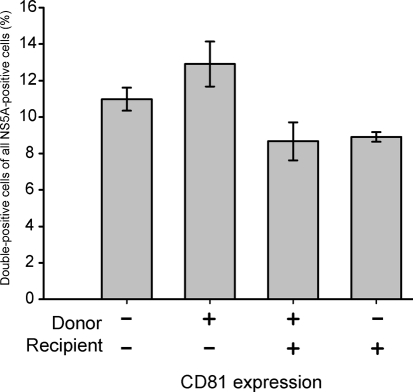
Expression of CD81, on either donor or recipient cells, is not necessary for cell-to-cell virus spread. Co-cultivations of different combinations of CD81-negative (Huh7L-#4) and -positive (Huh7L) donor cells (electroporated with JFH1_W529A_ RNA) and CD81-negative (Huh7L-#4) and -positive (Huh7L) recipient cells as shown were fixed and analysed by FACS. The frequency of cell-to-cell spread is presented as double-positive (NS5A/GFP) of all NS5A-positive cells (%).

The donor cells used in the above experiments express normal levels of CD81, which may somehow facilitate virus transmission into the recipient cells via a receptor-dependent route. To exclude this possibility, co-cultivation experiments using the CD81-negative cell line Huh7L-#4 as both donor and recipient were performed. As a control, the parental Huh7L cells were included. In all donor/recipient combinations no significant difference in cell-to-cell transmission was observed (one-way ANOVA, *P*>0.10) (Fig. 7), which rules out any possible contribution of CD81, present on either donor or recipient cells, in this process. Taken together, our data lead us to conclude that cell-to-cell transmission of HCVcc in culture does not involve CD81.

### Recipient to donor cell ratios determine levels of cell-to-cell transport

**Fig. 8. f8:**
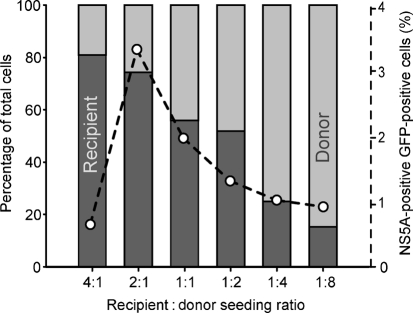
Donor to recipient cell ratio determines levels of cell-to-cell transport. JFH1_W529A_ RNA-electroporated donor Huh7L#4 cells were co-cultured with Huh7L#4–GFP cells at different initial seeding ratios as shown, and incubated for 4 days. The cells were fixed and analysed by flow cytometry, and the relative numbers of recipient (dark grey) and donor cells (light grey) as well as the rate of cell-to-cell transmission (broken line) were determined. The final ratio of the cells following incubation is presented as total cells (%), whereas the frequency of cell-to-cell transfer is shown as NS5A-positive cells of all GFP-positive cells (%).

To get a better understanding of the kinetics underlying cell-to-cell transport, various ratios of recipient to donor cells were used. Huh7L#4 cells electroporated with JFH1_W529A_ were co-cultured with the same cells expressing EGFP at different seeding ratios and the levels of cell-to-cell transport were determined by FACS. Interestingly, an increase in cell-to-cell transport was observed as the proportion of recipient cells was increased, reaching a maximum at an initial recipient to donor ratio of 2 : 1 (Fig. 8). However, the number of donor cells became a limiting factor at a ratio of 4 : 1. These results show that there is a clear correlation between the number of recipient cells and the frequency of cell-to-cell transmission.

## DISCUSSION

HCV, like other enveloped viruses, enters its target cells through a complex and not fully understood process involving virus interaction with several receptors and co-receptors. That said, the importance of the E2–CD81 interaction in HCV entry has been extensively explored. Based on our previous study with genotype 1a HCVpp (Owsianka *et al.*, 2006), we generated genotype 2a HCVcc mutant viruses defective in CD81 binding. The mutant particles bound host cells as efficiently as the WT virus, but failed to enter cells, suggesting that the defect lies at a post-binding stage, which is in keeping with the role of CD81 as a co-receptor (Cormier *et al.*, 2004). Together, our results confirm the critical role of the conserved E2 residues W529 and D535 in HCV infection, and provide a possible target for the future development of small-molecule entry inhibitors active against diverse HCV genotypes.

WT HCVcc was recently shown to be capable of direct cell-to-cell transmission in the presence of virus nAbs (Timpe *et al.*, 2008). We reasoned that using entry-defective cell-free mutant viruses without neutralizing agents would provide a more robust system to gain insights into the mechanism governing cell-to-cell transmission. Since the mutants described in this study are non-infectious, but otherwise comparable to the WT virus with respect to their replicative capacity and biophysical properties, they are ideally suited for this purpose. Co-cultivation of mutant-transfected cells with EGFP-expressing recipients resulted in a low level of cell-to-cell spread in culture. We further showed that HCV glycoproteins are essential for this mode of virus transmission.

Even though the interaction between mutant E2 and CD81 was undetectable in our assay, a low affinity HCV–CD81 interaction, either direct or facilitated by cell-to-cell contact, could account for the infected recipient cells. However, co-cultivation experiments in the presence of antibodies blocking the E2–CD81 interaction did not inhibit cell-to-cell transmission. To further establish the dispensability of CD81 in this entry process, we used several cell lines expressing either low or barely detectable levels of CD81. A clear correlation between the CD81 expression levels and susceptibility to WT virus infection was observed in them. We identified several cell lines that were refractive to infection with cell-free virus. In all these cell lines, whether expressing CD81 or not, and when used in any donor/recipient combination, cell-to-cell transmission of the mutant virus occurred at comparable levels. Together, these results unequivocally show that CD81 plays no role in the cell-to-cell spread of HCVcc.

Our results show that cell-to-cell transmission occurs at a relatively low frequency, generally requiring multiple HCV-positive donor cells to infect a single recipient cell. In contrast, the Timpe *et al.* (2008) study indicates one donor cell infecting up to five recipient cells, effectively amounting to cell-to-cell transmission being the predominant mode of virus spread in cell culture. If so, any replication-competent non-infectious virus capable of cell-to-cell transmission is expected to be stably maintained in cell culture. However, cells replicating our E2 mutant virus genomes were readily lost upon passaging. There are several possible explanations for these apparent discrepancies. Timpe *et al.* (2008) used WT virus-infected donor cells and concentrations of antibodies neutralizing less than 100 % of infection via the cell-free, CD81-mediated route. Also, the dye they used to label recipient cells [5′-chloromethylfluorescein diacetate (CMFDA)] is rapidly transported via gap junctions to adjacent cells (Barhoumi *et al.*, 1993; Bazou *et al.*, 2006; He *et al.*, 2007). This diffusion from the labelled recipient to the infected donor cells would result in gross overestimation of cell-to-cell transmission levels. Their study found HepG2, HeLa and HEK-293T cells supporting reduced levels of cell-to-cell transmission. It is likely that the CMFDA diffusion in these cells is impaired as they, unlike Huh7, contain low numbers of gap junctions or none at all (Carruba *et al.*, 2004; Clayton *et al.*, 2005; de Feijter-Rupp *et al.*, 1998; Stong *et al.*, 2006). In our co-culture system, we found no evidence for passive diffusion of EGFP from recipient to donor cells (Fig. 5a[Fig f5]).

A recent paper reported a CD81-deficient cell line, S29, in which cell-to-cell transmission of an infectious HCVcc was not observed, leading to the suggestion that CD81 is absolutely required for this process (Russell *et al.*, 2008). This observation conflicts with our data obtained using various independently generated CD81-deficient lines. Without direct comparison of the cell lines it is difficult to speculate, but the possible reasons for this anomaly may be a suboptimal donor to recipient cell ratio, or the lack of host factor(s) other than CD81 in this line. In this respect, S29 may prove a very useful cell line to identify host factors necessary for HCV cell-to-cell transmission.

HCV successfully evades the host-immune response despite the presence of nAbs. Direct cell-to-cell spread may be one way by which the virus is able to escape from nAbs and persist *in vivo*. As the mechanisms governing HCV cell-to-cell transmission are unknown, comparison with better-studied RNA viruses may provide some clues. Examples of cell-to-cell spread can be found in a number of viruses including rabies virus, human T-cell leukemia virus 1 and human immunodeficiency virus type 1. In all of these, viral spread via direct cell-to-cell transmission is protected from nAbs. In retroviruses, the induction of synapse-like structures facilitating cell-to-cell spread is triggered by the interaction of the viral glycoprotein (env) with specific host receptors. Blocking the env–cell receptor interaction abolishes cell-to-cell transmission (Jolly & Sattentau, 2004; Sherer *et al.*, 2007). Our results show that viral glycoproteins are essential for HCV cell-to-cell spread, but their interaction with CD81 is dispensable, so other receptors may be involved. It remains to be seen whether synapse-like structures govern this process in HCV. A considerable effort is currently being directed towards developing inhibitors of cell-free virus entry. In this respect, inhibitors targeting the HCV–CD81 interaction might be most effective in the transplant setting in preventing infection of the new organ by blood-borne virus, but less so in the context of ongoing chronic infection where direct cell-to-cell spread of the virus would remain unchecked. Therefore, for effective prevention and spread of infection, the cell-to-cell mode of virus transmission must also be considered as a target for antiviral development. The experimental systems described here will be useful for elucidating the processes involved in this mode of infection and their contribution to virus persistence and immune escape. Such information will prove important in the future design of inhibitors targeting HCV cell-to-cell transmission.

## Supplementary Material

[Supplementary Material]
