# Supplementary material for: CD81 is dispensable for hepatitis C virus cell-to-cell transmission in hepatoma cells
Source: J Gen Virol. 2009 Jan;90(Pt 1):48–58. doi: 10.1099/vir.0.006700-0 (PMC2885024; doi:10.1099/vir.0.006700-0)
Supplement: [Supplementary Material] [file supp_90_1_48__index.html]

 CD81 is dispensable for hepatitis C virus cell-to-cell transmission in hepatoma cells -- Witteveldt et al. 90 (1): 48 Data Supplement - Supplementary Material -- Journal of General Virology

## 

### CD81 is dispensable for hepatitis C virus cell-to-cell transmission in hepatoma cells, by J. Witteveldt, M. J. Evans, J. Bitzegeio, G. Koutsoudakis, A. M. Owsianka, A. G. N. Angus, Z.-Y. Keck, S. K. H. Foung, T. Pietschmann, C. M. Rice and A. H. Patel

*Journal of General Virology* vol. **90**, part 1, pp. 48 - 58

**Supplementary Material.** **Methods**

**Supplementary Fig. S1.** Determination of anti-CD81 neutralizing concentration, stability and effect of nAbs in co-culture experiments   
  
 [Single PDF file]  (245 KB)

  
  
