# Supplementary material for: CD81 is dispensable for hepatitis C virus cell-to-cell transmission in hepatoma cells
Source: J Gen Virol. 2009 Jan;90(Pt 1):48–58. doi: 10.1099/vir.0.006700-0 (PMC2885024; doi:10.1099/vir.0.006700-0)
Supplement: [Supplementary Material] [file supp_90_1_48__1.pdf]

Supplementary Material

Methods

Analysis of anti-CD81 mAb for neutralization experiments. The virus neutralizing potential of the anti-CD81 mAb was determined by pre-incubating Huh7 cells with different antibody concentrations for 30 min at 37 °C followed by infection with HCV strain JFH1. After 4 days of incubation, cells were harvested and the HCV RNA content measured by qRT-PCR as described in Methods section. As the cell-to-cell transmission experiments include a prolonged incubation time, we needed to determine the rate of cellular uptake and/or breakdown of the anti-CD81 mAb. For this, 1 μg ml^{-1} of the antibody was incubated with naïve Huh7 cells at 37 °C over a 24 h period. Culture medium samples were taken at regular intervals and the amount of anti-CD81 was determined by ELISA as follows. A serial dilution of the anti-CD81-containing cell medium was added to GST–CD81-coated wells. After incubation, the bound anti-CD81 was detected using anti-species IgG–HRP and the absorbance measured at 450 nm after addition of TMB substrate as described above.

Co-cultivation of cells in the presence of nAbs. For the co-culture experiments in the presence of anti-CD81, the recipient cells (Huh7–GFP) were pre-incubated for 30 min with 5 μg antibody ml^{-1} and added to the donor Huh7 cells electroporated with viral RNA. The anti-HCV E2 antibodies AP33 and CBH-5 at 50 and 5 μg ml^{-1}, respectively, were added to Huh7 cells electroporated with viral RNA 30 min before co-cultivating with Huh7–GFP cells. As a control, IgG1κ antibodies were used at 5 μg ml^{-1} (Sigma). The co-cultured cells received fresh medium containing the appropriate antibody at 24 h intervals until fixation.

Supplementary Fig. S1. Determination of anti-CD81 neutralizing concentration, stability and effect of nAbs in co-culture experiments. (a) Huh7 cells were pre-incubated with different concentrations of anti-CD81 mAb and infected with JFH1. At 4 days post-incubation, total RNA was isolated and the HCV RNA levels measured by qRT-PCR. Virus infection was completely abrogated when cells were pre-incubated at a concentration of 5 μg ml^{-1} or higher. (b) The possible cellular uptake of anti-CD81 mAb was investigated by incubating Huh7 cells with 1 μg antibody ml^{-1} over a 24 h period. Medium samples were collected at different time points (t=0–24, as indicated) and the amount of anti-CD81 was determined by ELISA. There was no detectable depletion of antibody, other than an initial decrease, probably due to binding to cellular CD81. (c) Cell-to-cell transport of HCV in the presence of neutralizing concentrations of anti-CD81 or anti-E2 antibodies as indicated. Cells expressing both NS5A and EGFP are indicated by arrows. Immunofluorescence analysis showed that despite the presence of nAbs that block the E2–CD81 interaction, similar rates of cell-to-cell transport were found, indicating that the E2–CD81 interaction is not necessary for the direct transmission of HCV.

Witteveldt, J., Evans, M. J., Bitzegeio, J., Koutsoudakis, G., Owsianka, A. M., Angus, A. G. N., Keck, Z.-Y., Foung, S. K. H., Pietschmann, T., Rice, C. M. and Patel, A. H. (2009). CD81 is dispensable for hepatitis C virus cell-to-cell transmission in hepatoma cells. *J Gen Virol* **90**, 48–58.

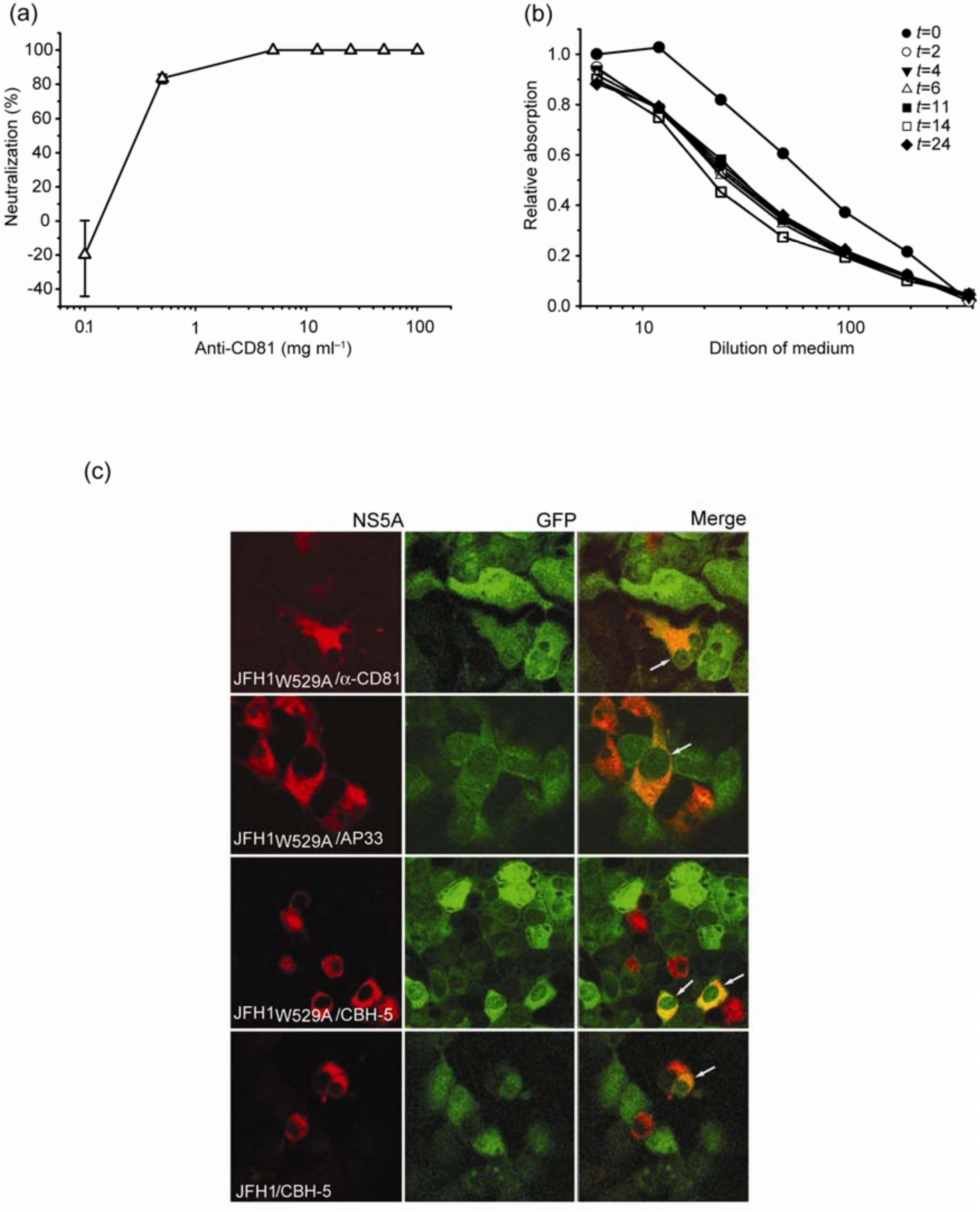

Supplementary Fig. S1. Determination of anti-CD81 neutralizing concentration, stability and effect of nAbs in co-culture experiments. (a) Huh7 cells were pre-incubated with different concentrations of anti-CD81 mAb and infected with JFH1. At 4 days post-incubation, total RNA was isolated and the HCV RNA levels measured by qRT-PCR. Virus infection was completely abrogated when cells were pre-incubated at a concentration of 5 μg ml^{-1} or higher. (b) The possible cellular uptake of anti-CD81 mAb was investigated by incubating Huh7 cells with 1 μg antibody ml^{-1} over a 24 h period. Medium samples were collected at different time points (t=0–24, as indicated) and the amount of anti-CD81 was

determined by ELISA. There was no detectable depletion of antibody, other than an initial decrease, probably due to binding to cellular CD81. (c) Cell-to-cell transport of HCV in the presence of neutralizing concentrations of anti-CD81 or anti-E2 antibodies as indicated. Cells expressing both NS5A and EGFP are indicated by arrows. Immunofluorescence analysis showed that despite the presence of nAbs that block the E2–CD81 interaction, similar rates of cell-to-cell transport were found, indicating that the E2–CD81 interaction is not necessary for the direct transmission of HCV.
